# A Rare Case of Primary Cutaneous Epstein-Barr Virus-Positive T Follicular Helper Cell Lymphoma

**DOI:** 10.7759/cureus.96925

**Published:** 2025-11-15

**Authors:** Osamu Okamoto, Yuzo Oyama, Rika Maruyama, Kentaro Nagamatsu, Morishige Takeshita

**Affiliations:** 1 Division of Dermatology, Oita City Medical Association’s Almeida Memorial Hospital, Oita, JPN; 2 Department of Diagnostic Pathology, Faculty of Medicine Oita University, Yufu, JPN; 3 Division of Hematology, Oita City Medical Association’s Almeida Memorial Hospital, Oita, JPN; 4 Division of Diagnostic Pathology, Saiseikai Yahata General Hospital, Kitakyushu, JPN

**Keywords:** cd4-positive, epstein-barr virus, primary cutaneous, progressive, t cells, t follicular helper cell lymphoma

## Abstract

We herein report a case of Epstein-Barr virus-positive primary cutaneous T follicular helper cell lymphoma (EBV-positive pcTFHL) in an elderly patient. The patient presented with multiple erythemas and cutaneous and subcutaneous nodules on the legs without internal organ involvement. Histology demonstrated diffuse infiltrates of atypical small- to medium-sized lymphoid cells in the dermis and subcutis associated with exocytosis in the epidermis and extensive necrosis in the deep dermis. Immunohistochemistry demonstrated that the atypical lymphoid cells were CD3- and CD4-positive T cells and expressed TFH cell markers. The CD4-positive atypical lymphoid cells were also diffusely positive for EBV byin situ hybridization, confirming the diagnosis of EBV-positive pcTFHL. The clinical course was progressive: large multiple cutaneous ulcers developed, and the patient died of fibrinous and organizing pneumonia due to COVID-19. The total clinical course was two years and two months after the onset of the skin rashes. This is the third case of EBV-positive pcTFHL that showed EBV infection exclusively in CD3- and CD4-positive T cells and had a lethal progressive clinical course.

## Introduction

A new disease category of nodal T follicular helper (TFH) cell lymphoma (nTFHL) was created, comprising three types: angioimmunoblastic (AI)-type, follicular (F)-type, and not otherwise specified (NOS) in the World Health Organization Classification of Haematolymphoid Tumors (WHO-HAEM5) [1]. The TFH phenotype is defined to express TFH markers of CD10, BCL6, PD1, CXCL13, and ICOS [2]. These nTFHLs are closely related to Epstein-Barr virus (EBV) infection [3,4], and EBV is detected in non-neoplastic B cells. This newly created entity of nTFHL concerns only TFHLs with nodal lesions, and a distinct classification of a primary cutaneous TFHL (pcTFHL) does not exist. Therefore, presently, pcTFHL has to be classified into a “peripheral T-cell lymphoma, not otherwise specified (PTCL-NOS)”. At present, the term "pcTFHL" is provisionally used to represent the specific phenotype of this unclassified disease entity. 

PTCL is an entity in which lymphoma cells originated from mature T cells or natural killer (NK) cells [2]. This is an uncommon entity that accounts for approximately 7% of the overall lymphoma population and generally has an aggressive nature. PTCL can be roughly classified into the following types: cutaneous (e.g., mycosis fungoides (MF), Sézary syndrome, and pcPTCL-NOS), extranodal (e.g., extranodal NK/T cell lymphoma), anaplastic large cell lymphomas, leukemic (e.g., adult T cell lymphoma/leukemia and aggressive NK cell leukemia), and enteropathy-associated [5]. According to the present knowledge, pcPTCL with TFH phenotypes is largely composed of CD4-positive small/medium-sized T-lymphoproliferative disorder (T-LPD) and MF [6,7].

Recently, Chiang et al. reported two cases of elderly patients with EBV- and CD4-positive pcTFHL with a lethal progressive clinical course [8]. Wang et al. reported 23 cases of CD4- and TFH-positive pcTFHL [9]; however, the phenotypes varied with regard to B cell involvement and EBV infection [9]. Thus, presently, clinicopathological information regarding pcTFHL with EBV infection in T cells is quite limited [8,9]. We also experienced the case of an elderly patient with EBV and CD4-positive pcTFHL with a lethal progressive clinical course. In this report, we attempted to establish knowledge about EBV-positive pcTFHL among groups with EBV-positive TFHL and T-LPD. 

## Case presentation

**Figure 1:**
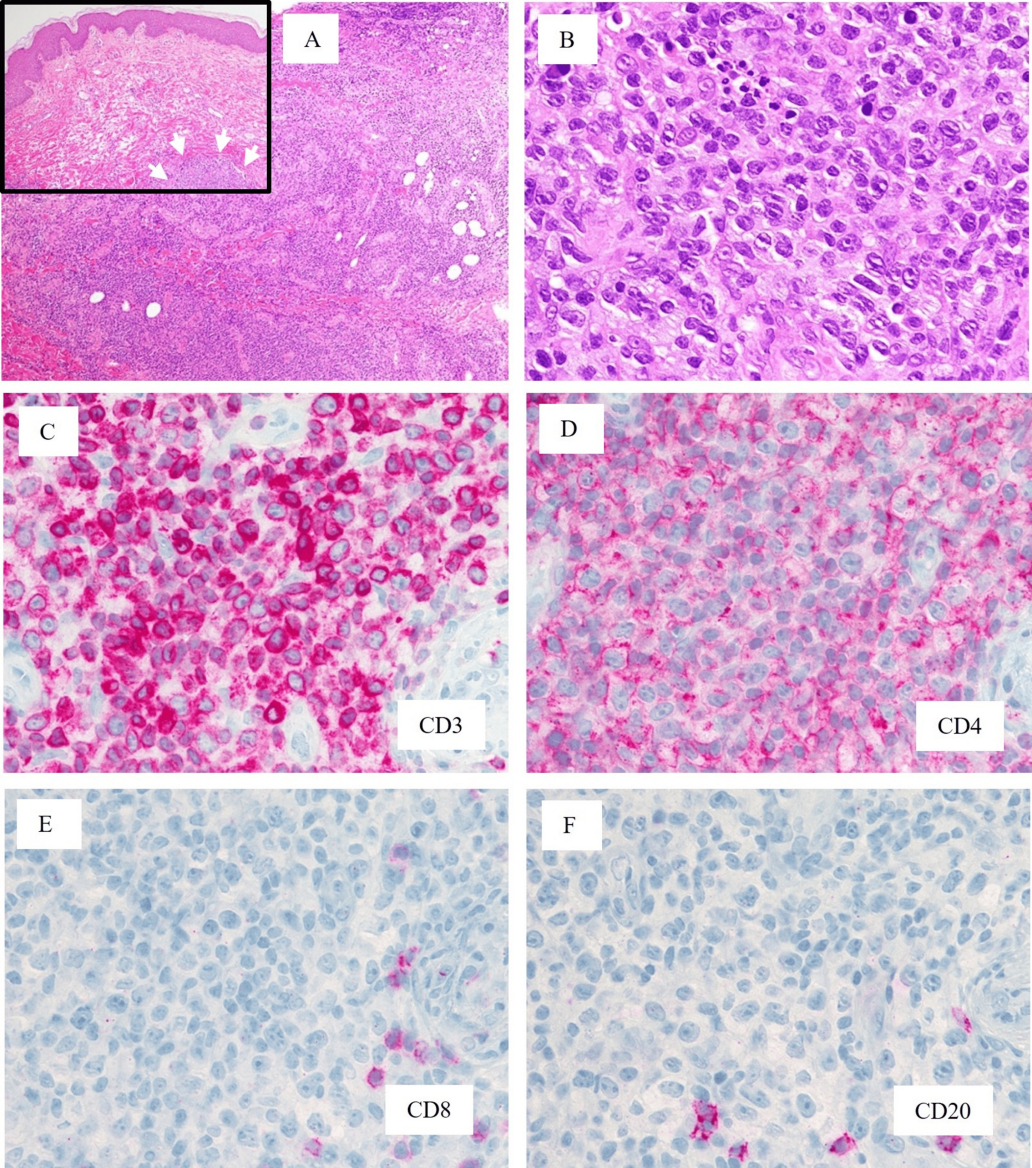
Histology and immunophenotypes of the first biopsy performed in a clinic in February in the second year of onset. A, B: H&E staining; A: Low-power view (×40), diffuse infiltration of neoplastic cells is observed in the deep dermis. Upper inset: superficial lesion, no epidermal changes were observed (×40). The most superficial part of the neoplastic cell infiltration is indicated by arrows. B: High-power view of the infiltrating neoplastic cells (×400). C-F: Immunostaining (×400). C: CD3; D: CD4; E: CD8; F: CD20. Magenta indicates positive staining.

A 75-year-old man presented with scaly parapsoriasis-like plaques on the lower legs in May, in the first year of onset (11 months prior to the patient’s first visit to our division). The patient had been treated with bicalutamide for prostate cancer with bone metastases for six years. The rashes became erythematous in August, and because multiple nodules appeared in the legs in the second year of onset (not shown), the patient visited a dermatological clinic, and a skin biopsy was performed. Hematoxylin and eosin (H&E) staining showed diffuse infiltration of medium-sized atypical lymphoid cells with deeply stained nuclei and dispersed mitotic figures in the deep dermis and subcutis (Figures 1A, 1B). Microabscesses were not found in the epidermis of the biopsied sample (Figure 1A, inset). The atypical cells were CD45 (leukocyte common antigen, LCA)-, CD45RO (UCHL-1)-, CD3-, and CD4-positive (Figures 1C, 1D) and CD8- and CD20-negative (Figures 1E, 1F). Because MF was suspected, the patient was transferred to our division in March.

**Figure 2:**
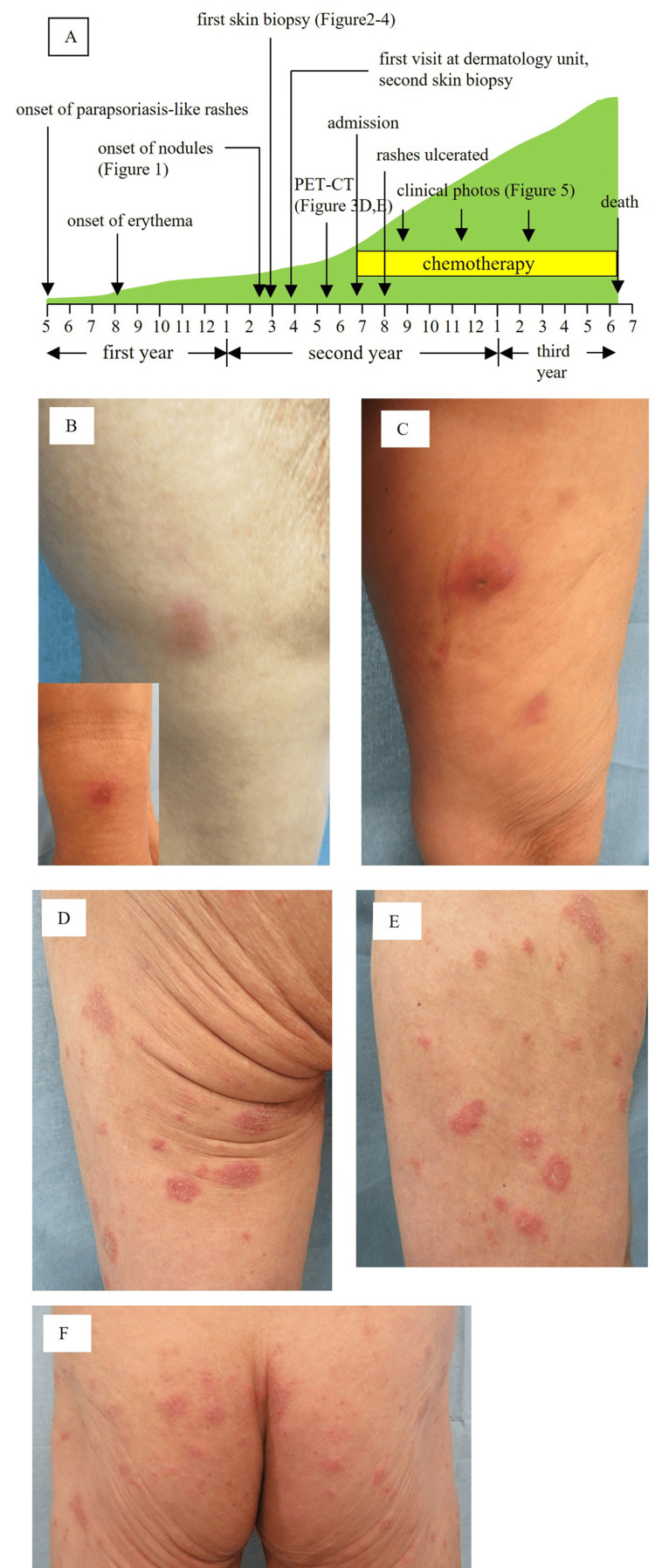
Clinical course of the case and appearances A: The total clinical course of the current case. The horizontal bar indicates time. Time is expressed as months (one to 12), and the years are indicated at the bottom of the panel. The light-green area indicates the area of skin rashes. The yellow bar indicates the duration of chemotherapy. B-F: Clinical appearance of the current case at the first visit to our division in March, in the second year of onset. B: Nodules in the right knee joint and distal aspect of the right lower leg (inset). C: Left upper leg. Erythemato-plaques on the upper leg (D), lower leg (E), and the buttocks (F).

**Figure 3:**
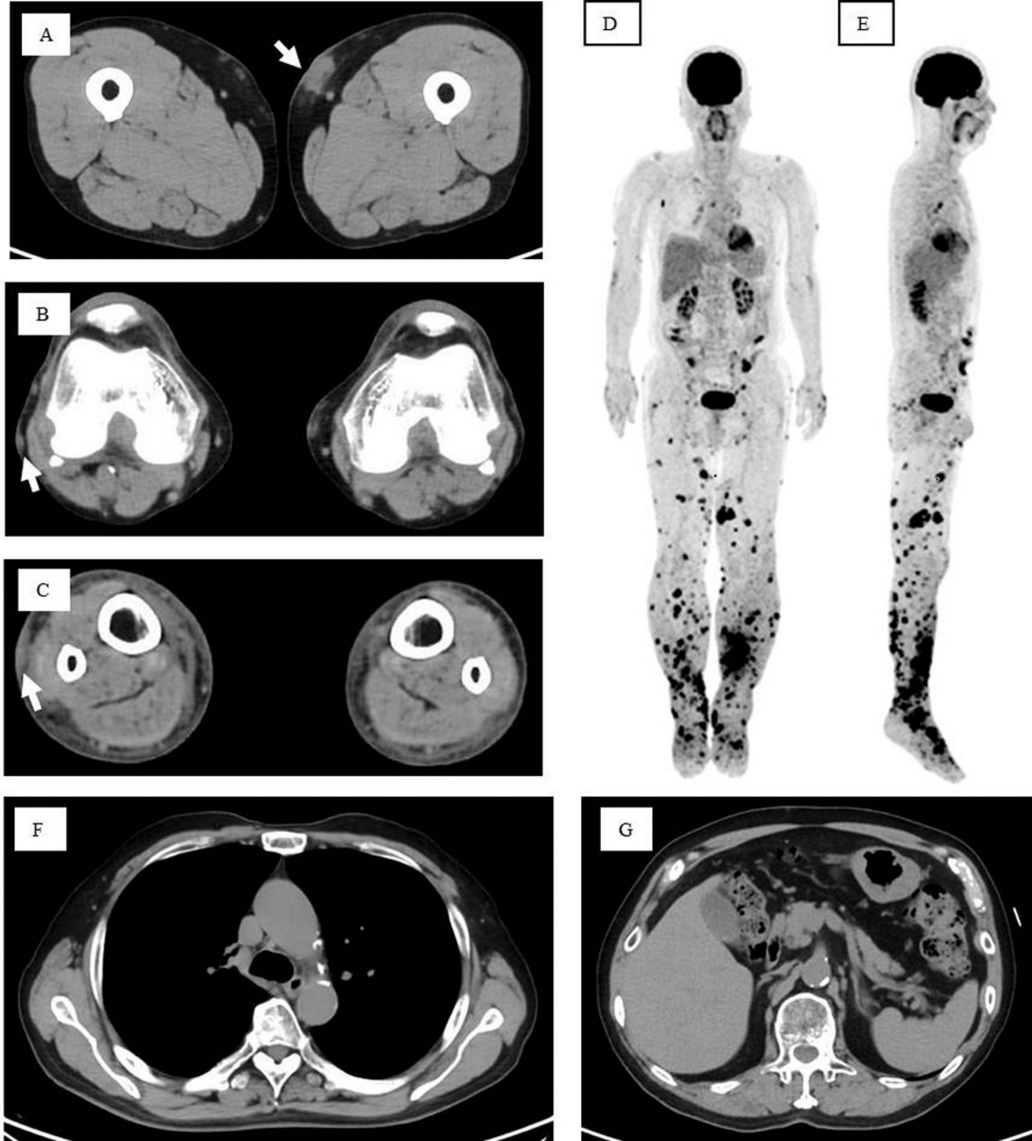
Imaging analyses A-C: Computed tomography (CT) images of the legs in March in the second year of onset. Tumor in the left upper leg (A), right knee joint (B), and distal aspect of the right lower leg (C). The tumors are indicated by the white arrows. D, E: Positron emission tomography-CT (PET-CT) images in May in the second year of onset. Anterior (D) and side (E) aspects of PET-CT images. The substantial uptake of ^18^F-fluorodeoxyglucose (FDG) is distributed on the surface of both legs (compare anterior and side aspect images); conversely, FDG accumulation in the brain and other internal organs appears physiological. F, G: Chest and abdominal CT images showing no pathological lymph node enlargement or internal organ lesions. Slices at the pulmonary hilus (F) and beginning of the celiac trunk (G) were chosen as representatives.

The clinical course of the patient is summarized in Figure 2A. Upon examination, the patient’s temperature was 36.4℃, several indurated cutaneous nodules with surface erythema were scattered on both legs (Figures 2B, 2C), and erythemato-plaques were scattered on the upper leg (Figure 2D), lower leg (Figure 2E), and buttocks (Figure 2F). The results of the blood examination are presented in Table 1. EBV DNA was detected in the blood at a high titer (5.96 log IU/ml: 1.8-2.3×10^5^ copies/μg), and anti-human T-lymphotropic virus (HTLV)-1 antibody was negative. The white blood cell (WBC) count was 4,690/μl (lymphocytes 16.0%, abnormal lymphocytes 0%), total bilirubin: 0.8 mg/dl, lactate dehydrogenase (LDH): 192 U/l, and soluble interleukin-2 receptor (sIL-2R): 719 U/ml. No increase in the levels of the other liver enzymes was observed. Imaging analyses using computed tomography (CT) revealed skin lesions (Figures 3A-3C) with no apparent internal lesions noted (Figures 3F, 3G). Positron emission tomography-CT performed in May demonstrated numerous instances of the uptake of ^18^F-fluorodeoxyglucose on the surface of both legs, while no abnormal uptake in other organs was observed (Figures 3D, 3E).

**Table 1:** The patient's blood analysis data Units and normal ranges are shown in parentheses. Marks “-” indicate negative findings. H and L denote abnormal high and low values, respectively.

Test	Reference range	Value
Total protein	6.4-8.4 g/dl	7.6
Albumin	3.6-5.2 g/dl	4.9
Albumin/globulin (A/G) ratio	1.3-2.0	1.8
Total bilirubin	0.2-1.2 mg/dl	0.8
Hemoglobin A1c	4.6-6.2 %	6.2
Urea nitrogen (UN)	6-22 mg/dl	13
Creatinine	0.6-1.1 mg/dl	0.69
Uric acid	2.5-7.0 mg/dl	6
Glucose	70-109 mg/dl	114 (H)
Total cholesterol	120-219 mg/dl	159
Neutral fat	30-149 mg/dl	137
High-density lipoprotein (HDL) cholesterol	40 mg/dl ≤	51
Low-density lipoprotein (LDL) cholesterol	70-139 mg/dl	80
Aspartate aminotransferase (AST)	13-33 U/l	22
Alanine aminotransferase (ALT)	6-30 U/l	16
Lactose dehydrogenase (LDH)	106-211 IU/l	192
Alkaline phosphatase (ALP)	38-113 U/l	59
Cholinesterase (ChE)	229-521 U/l	314
Gamma-glutamyl transpeptidase (γ-GTP)	16-73 U/l	19
Creatine kinase (CK)	56-244 U/l	111
Amylase	39-115 U/l	73
Sodium (Na)	135-146 mEq/l	141
Potassium (K)	3.5-5.0 mEq/l	4
Chloride (Cl)	96-108 mEq/l	104
Calcium (Ca)	8.5-10.0 mg/dl	9.7
C-reactive protein (CRP)	0-0.23 mg/dl	0.1
Soluble interleukin-2 receptor (sIL-2R)	145-519 U/ml	719 (H)
Immunoglobulin G (IgG)	870-1,700 mg/dl	1,186
Immunoglobulin A (IgA)	110-410 mg/dl	199
Immunoglobulin M (IgM)	35-220 mg/dl	91.9
Rapid plasma reagin card test (RPR)	-	-
*Treponema pallidum* hemagglutination test (TPHA)	-	-
Ferritin	13-277 ng/ml	25.6
Hepatitis B virus S antigen	-	-
Anti-hepatitis C virus antibody	-	-
Anti-human T-lymphotropic virus (HTLV)-1/2 antibody	-	-
White blood cell (WBC)	3,900-9,800 /μl	4,690
Differential counts of WBC		
‧ Blast	0%	0
‧ Promyelocyte	0%	0
‧ Myelocyte	0%	0
‧ Metamyelocyte	0%	0
‧ Stab cell	0-6 %	0
‧ Segmented neutrophil	32-73 %	69
‧ Lymphocyte	18-59 %	16 (L)
‧ Monocyte	0-8 %	7
‧ Eosinophil	0-7 %	7
‧ Basophil	0-2 %	1
‧ Atypical lymphocyte	0-2 %	0
Red blood cell (RBC)	4.2-5.7×10^6^ /μl,	4.52
Hemoglobin	13.5-17.6 g/dl	12.6 (L)
Hematocrit	39.8-51.8 %	38 (L)
Mean cellular volume (MCV)	82.7-101.6 fl	84.1
Mean corpuscular hemoglobin (MCH)	28-34.6 pg	27.9 (L)
Mean corpuscular hemoglobin concentration (MCHC)	31.6-36.6 %	33.2
Platelet	131-362×10^3^/μl	161
Prothrombin time	10.5-13.5 second	13.1
Prothrombin time-international normalized ratio (PT-INR)	0.85-1.15	0.99
Activated partial thromboplastin time (APTT)	24-39 second	30.6
Fibrinogen	200-400 mg/dl	301
Fibrin degradation product (FDP)	< 5.0 μg/ml	3.1
Serum protein fractionation		
‧ Albumin	55.8-66.1 %	58.7
‧ α1 globulin	2.9-4.9 %	3.5
‧ α2 globulin	7.1-11.8 %	9.8
‧ β1 globulin	4.7-7.2 %	6.6
‧ β2 globulin	3.2-6.5 %	5.1
‧ γ globulin	11.1-18.8 %	16.3
Epstein-Barr virus (EBV)-DNA	(-) log IU/ml	5.96 (H)

**Figure 4:**
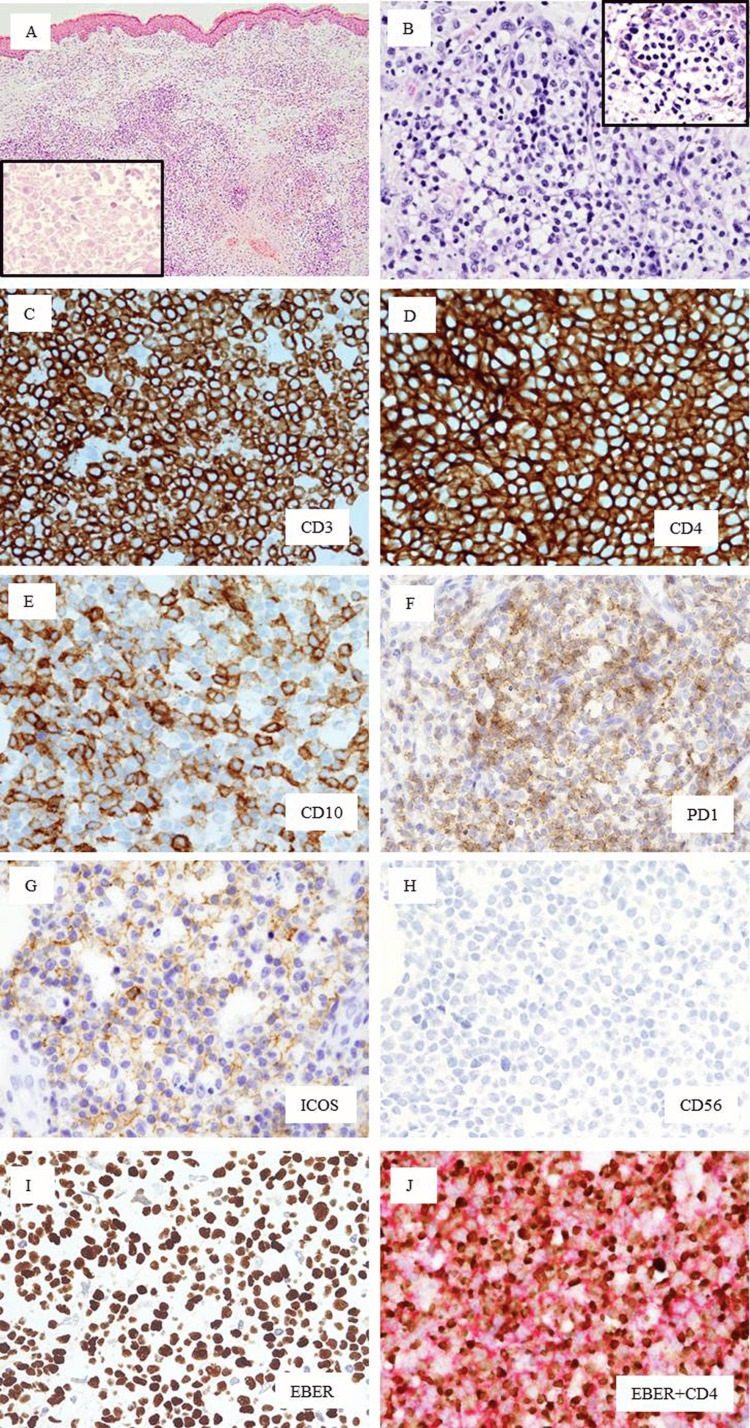
Histology and immunophenotypes of the current case A, B: H&E staining; A: Low-power view (×40), diffuse infiltration of neoplastic cells is observed in the dermis. Inset: Coagulative necrotic area in the deep dermis (×400). B: High-power view of the infiltrating neoplastic cells. Numerous small- to medium-sized lymphoid cells with hyperchromatic nuclei and large cells with clear cytoplasm are observed (×400). Inset: Exocytosis of lymphocytes in the epidermis (×400). C-H: Immunostaining (×400). C: CD3; D: CD4, E: CD10, F: PD1, G: ICOS, H: CD56. Brown indicates positivity. I: EBER-ISH. Brown indicates positivity. J: Double staining of EBER-ISH and CD4 (×400). Brown indicates positivity for EBER-ISH, and magenta indicates positivity for CD4. EBER: Epstein–Barr virus–encoded RNA; ISH: in situ hybridization

An additional biopsy of the skin nodule was performed, and H&E staining showed diffuse or partial nodular infiltration of small- or medium-sized atypical lymphoid cells with clear cytoplasm, with scattered large lymphoid cells in the dermis and subcutis (Figures 4A, 4B). Eosinophils and plasma cells were few in number. Coagulation necrosis was also observed in the subcutis (Figure 4A inset). In the epidermis, exocytosis of lymphocytes resembling Pautrier’s microabscesses was observed (Figure 4B inset). The immunohistochemistry results are shown in Figures 4C-4J and Table 2. The atypical lymphoid cells were diffusely positive for CD3 (Figure 4C), CD4 (Figure 4D), CD30, MIB1 (60%), and TFH markers of CD10 (Figure 4E), PD1 (Figure 4F), and ICOS (Figure 4G) but negative for BCL6, CD8, CD56 (Figure 4H), and cytotoxic markers (CMs) of TIA1 and granzyme B (Table 2). Some CD20-positive B lymphocytes were scattered throughout the lesions (data not shown). Some nests of CD21-positive dendritic cells were also detected (data not shown). Numerous EBV-positive lymphoid cells were detected by in situ hybridization (ISH) (Figure 4I), and double staining confirmed that these EBV-positive cells were positive for CD3 and CD4 (Figure 4J) and negative for CD20 (data not shown). Since latent membrane protein-positive and EBV nuclear antigen 2-negative lymphocytes were scattered, EBV latency type II was noted. Finally, EBV-positive pcTFHL was considered.

**Table 2:** Immunohistochemical marker profiles of the tumor cells “+” indicates positive staining, and “-” indicates negative staining.

Markers	
CD3	+
CD4	+
CD5	+
CD8	-
CD30	+
CD10	+
BCL6	-
PD1	+
ICOS	+
CXCL13	-
CD56	-
TIA1	-
Granzyme B	-
CD20	-
CD79a	-
FOXP3	-
MIB1	+
EBER-ISH	+
LMP1	+
EBNA2	-
CD25	+
c-MYC	+
CCR4	+

**Figure 5:**
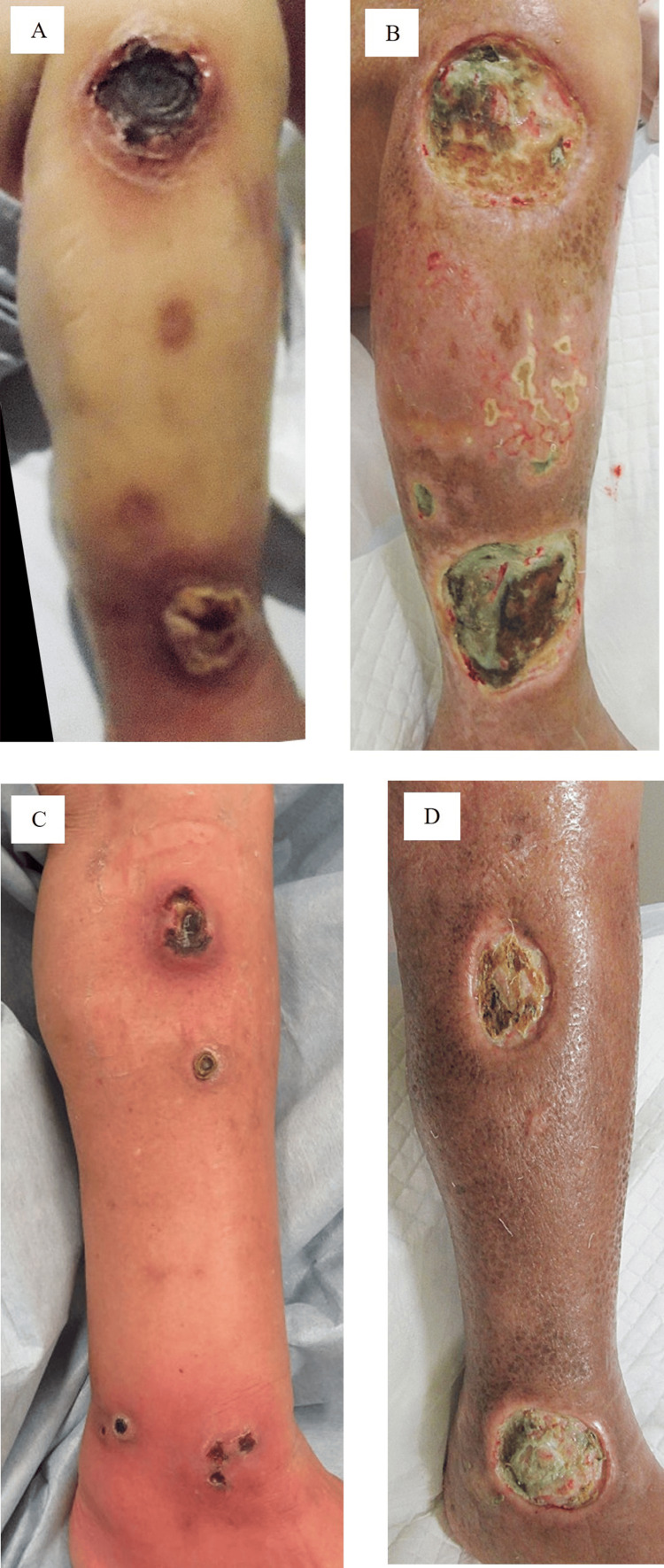
Disease progression in the following years A, B: Lesions in the right lower leg and C, D: lesions in the left lower leg. A: November in the second year and B: February in the third year of onset. C: August in the second year and D: February in the third year of onset. Note that the main ulcers increased in size and were infected by *Pseudomonas aeruginosa* (light-green hue on the ulcers).

The patient was transferred and admitted to the hematology division in June in the second year of onset (Figure 2A), and no invasion by atypical lymphoid cells was confirmed via bone marrow biopsy. A+CHP (Adcetris+cyclophosphamide, doxorubicin hydrochloride, prednisolone) regimen (brentuximab vedotin 1.8 mg/kg, cyclophosphamide 750 mg/m^2^, adriamycin 50 mg/m^2^, prednisolone 80 mg/ body) was initiated in June, but after a total of seven courses of chemotherapy, the nodules increased in size and were ulcerated from August (Figures 5A, 5C). Therefore, chemotherapy was changed to romidepsin injection (14 mg/m²) in November, yet nodules and ulcers increased in size. Then mogamulizumab injection (1 mg/kg) was initiated, and radiotherapy was combined (52.3 Gy/23 fractions) in December. However, because systemic drug eruption due to mogamulizumab developed in March in the third year of onset, oral tucidinostat (40 mg/body, twice/week) was finally initiated. Nevertheless, the ulcers still increased (Figures 5B, 5D), moderate liver dysfunction appeared in May, and the patient was finally infected with COVID-19 and died of acute fibrinous and organizing pneumonia in mid-June (Figure 2A).

The final blood analysis results were as follows: WBC count 24,580/μl (neutrophils 98.7%, lymphocytes 0.7%, abnormal lymphocytes 0%), C-reactive protein (CRP) 8.67 mg/dl, potassium (K) 7.9 mEq/l, LDH: 221 U/l, sIL-2R: 7,530 U/ml, pH: 7.282, pCO₂: 50.2 mmHg. Repeated imaging analyses did not reveal any pathological systemic lymphadenopathies during the clinical course. The total clinical course was two years and two months from the onset of parapsoriasis-like skin rashes and 15 months after the diagnosis of lymphoma.

## Discussion

The current TFHL case is exceptional in that it was a primary cutaneous EBV infection found in CD3- and CD4-positive T cells, and the number of EBV-infected lymphoid cells was numerous. The current case highlights these exceptional characteristics and the progressive nature of EBV-positive pcTFHL. Given the age distribution, characteristic tumor immunophenotypes, and progressive clinical behaviors, this pcTFHL case expands our knowledge of EBV-related T-LPDs and provides clinical information regarding the special TFHL subset.

The most infected cells by EBV are B cells, while occasionally a small fraction of NK/T cells are infected [10]. After infection of B cells, potent host immune responses with cytotoxic T cells and NK cells are triggered, and most infected B cells are eliminated [10]. On the other hand, in cases of NK/T cell infection by EBV, the elimination of the infected cells occasionally fails, probably because of dysfunction of cytotoxic T cells [11], and some cases proceed to EBV-related LPDs. The elimination of the infected cells would not occur evenly, because the causative cell types depend on the types of EBV-related LPDs [12]. A similar elimination failure may have occurred in the development of pcTFHL in the current case; however, the mechanisms underlying the selective expansion of the infected cell type (CD4-positive T cells in the current case) are not known.

The erythema-plaque-tumor course in the current case indicated a similarity to MF; therefore, MF was a possible diagnostic candidate. It is known that MF has TFH phenotypes and is, to various extents, associated with EBV infection according to polymerase chain reaction analyses of tissue extracts [7,13]. However, the etiologic role of EBV in MF is contradictory, and in the majority of reports, EBV-positive lymphoid cells were undetectable or few by ISH [13-15]. Similarly, in one cumulative report, the EBV viral load in the peripheral blood was low (10-1,904 copies/μg, median 13.5 copies/μg) [13]. In contrast, the current case demonstrated a high viral load in the peripheral blood (5.96 log IU/ml: 1.8-2.3×10^5^ copies/μg), and numerous EBV- and CD4-positive lymphoid cells were observed in the skin lesion. The unusually extensive EBV infection profiles in the current case clearly indicate a fundamental role of EBV in the development of the lesion. Therefore, the diagnosis of MF in the current case was not likely.

Wang et al. reported 23 cases of CD4- and TFH-positive pcTFHL [9]. In the publication, EBV-positive lymphocytes were detected in six of 23 pcTFHL cases (26%), and four had associated EBV-positive diffuse large B-cell lymphoma or EBV-positive B-LPDs, while two died of disease at 12 and 48 months. However, phenotypes similar to the current case (CD4- and EBV-positive PTCL without B cell involvement) were not found. In contrast, Chiang presented two elderly cases of EBV-positive pcTFHL [8], and the lesions in both cases initially showed papules and plaques; one developed multiple skin lesions followed by lymphadenopathy, and another developed nodal and extranodal lesions. EBV infection was confirmed in neoplastic CD3-positive T cells by double staining, and finally, the two patients died of the disease within six months.

The current case presented with skin lesions and immunophenotypes similar to those reported by Chiang et al. [8], although the lesions in the current case were limited to the skin. Taken together, this is the third case of pcTFHL with neoplastic EBV- and CD4-positive T cells. In addition, Zhang et al. reported two elderly EBV-positive nTFHL cases with extranodal involvement, showing a lethal progressive clinical course at eight and eleven months [16]. These reports suggest that EBV-positive neoplastic T cells in TFHLs caused a lethal progressive clinical course. Further cumulative studies are necessary to confirm the clinicopathological features of EBV-positive pcTFHLs.

CD10, BCL6, PD1, CXCL13, and ICOS are TFH cell markers, and at least two, or preferably three, markers are required for the identification of TFH cells [2]. Three markers (CD10, PD1, and ICOS) were positive in the current case; thus, the tumor cells were identified as TFH cells. On the other hand, TIA1 and granzyme B are CM markers, and CD56 is an NK cell marker. Therefore, positivity for these markers indicates NK cell- or cytotoxic T cell-derived TCLs, and thus TFHLs can be excluded. An extranodal EBV-positive NK- and T-cell lymphoma (NKTCL) is a representative EBV-related PTCL and was therefore a diagnostic candidate. Extranodal EBV-positive NKTCL mainly demonstrates a CD4- and CD8-negative, CD56-positive, and rarely a CD8-positive phenotype [1, 17]; nevertheless, skin invasion by CD4-positive extranodal NKTCL has been reported in an elderly patient [18]. This case demonstrated a CD8- and CD20-negative phenotype; however, the patient was positive for CD56 or CMs, which are indispensable features of extranodal EBV-positive NKTCL [1,17]. Hence, the lack of expression of CD56 and CM in our patient excluded a diagnosis of EBV-positive NKTCL. Hydroa vacciniforme-like LPD (HV-LPD) and severe mosquito bite allergy (SMBA), are well-known cutaneous EBV-related LPDs in childhood and rare in adults [1,19]. The infiltrating lymphoid cells show varying expression of CD4 and CD8, but the expression of CMs or CD56 is invariable [1,19]. This CM positivity excludes the diagnosis of HV-LPD and SMBA in the current case.

The A+CHP regimen is a standard regimen for treatment of CD30-positive PTCLs [20]; however, when the response appears poor, salvage therapies including mogamulizumab, romidepsin, and tucidinostat would be added. In the previous relevant reports, Chiang et al. used the COP (cyclophosphamide, vincristine, and prednisolone) regimen for case 1 and the SMILE (dexamethasone, methotrexate, ifosfamide, L-asparaginase, and etoposide) regimen for case 2 [8], while Zhang et al. used the CHOP (cyclophosphamide, doxorubicin, vincristine, and prednisolone) regimen for cases 1 and 2, which showed lethal outcomes [16]. In the current case, because the clinical course deteriorated during the A+CHP regimen, we tried additional treatments, but the disease activity did not regress. Moreover, radiation therapy did not demonstrate an apparent effect. Taken together, the earlier initiation of chemotherapy could have, to some extent, delayed the disease progression. However, the patient’s death due to the disease would have been inevitable. Thus, it is recognized that the pcTFHL has an aggressive phenotype.

## Conclusions

We reported an elderly case of pcTFHL with numerous EBV- and CD4-positive neoplastic T cells. This is an exceptional TFHL in that it is primarily cutaneous and that EBV infection was exclusively found in T cells. It is highly speculated that EBV-positive pcTFHL belongs to a rare group, occurring in older patients and representing a lethal progressive clinical course. 

The pcTFHL has several features similar to MF; therefore, some relevant cases might be recognized as MF or pcPTCL-NOS. Close examinations for EBV in such cases may provide an opportunity to discover unusual pcPTCL phenotypes. The accumulation of clinicopathological knowledge about such unusual cases will provide a more practical understanding of the phenotype-prognostic relationships of pcPTCLs.
